# Development and validation of machine learning models for venous thromboembolism risk assessment at admission: a retrospective study

**DOI:** 10.3389/fcvm.2023.1198526

**Published:** 2023-08-29

**Authors:** Wenbo Sheng, Xiaoli Wang, Wenxiang Xu, Zedong Hao, Handong Ma, Shaodian Zhang

**Affiliations:** ^1^ Research and Development Department, Shanghai Synyi Medical Technology Co., Ltd., Shanghai, China; ^2^ Pudong Institute for Health Development, Shanghai, China; ^3^ Division of Medical Affairs, Shanghai Tenth People's Hospital, Shanghai, China

**Keywords:** venous thromboembolism, risk assessment model, machine learning, predictive modeling, risk stratification

## Abstract

**Introduction:**

Venous thromboembolism (VTE) risk assessment at admission is of great importance for early screening and timely prophylaxis and management during hospitalization. The purpose of this study is to develop and validate novel risk assessment models at admission based on machine learning (ML) methods.

**Methods:**

In this retrospective study, a total of 3078 individuals were included with their Caprini variables within 24 hours at admission. Then several ML models were built, including logistic regression (LR), random forest (RF), and extreme gradient boosting (XGB). The prediction performance of ML models and the Caprini risk score (CRS) was then validated and compared through a series of evaluation metrics.

**Results:**

The values of AUROC and AUPRC were 0.798 and 0.303 for LR, 0.804 and 0.360 for RF, and 0.796 and 0.352 for XGB, respectively, which outperformed CRS significantly (0.714 and 0.180, *P* < 0.001). When prediction scores were stratified into three risk levels for application, RF could obtain more reasonable results than CRS, including smaller false positive alerts and larger lower-risk proportions. The boosting results of stratification were further verified by the net-reclassification-improvement (NRI) analysis.

**Discussion:**

This study indicated that machine learning models could improve VTE risk prediction at admission compared with CRS. Among the ML models, RF was found to have superior performance and great potential in clinical practice.

## Introduction

Venous thromboembolism (VTE) includes pulmonary embolism (PE) and deep vein thrombosis (DVT), two clinical manifestations of the disease at different stages and locations. As a common complication and adverse event during hospitalization, VTE is one of the leading causes of preventable hospital death (presented with PE) and increased length of stay (1, 2). In a Chinese multicenter study between 2007 and 2016, the age- and sex-adjusted hospitalization rates of VTE patients increased from 3.2 to 17.5 per 100,000 population, and the hospitalization mortality rates decreased from 4.7% to 2.1% (3). The studies of diagnosis, therapy, and prevention have been rapidly developed recently (4, 5).

VTE risk is constantly changing during patients' hospitalization and may be triggered by the clinical intervention of surgery and anesthesia. Throughout the whole cycle of dynamical assessments, the time point of admission is of great importance for VTE management. Performing pre-test assessment at admission could promote risk stratification and follow-up decision-making, and early screening and prophylaxis could effectively reduce the incidence (6). Currently, a great number of VTE risk-assessment models (RAMs) have been proposed, mainly including a list of clinical factors and corresponding scores. There have been several studies focusing on the validation of RAMs at admission to guide early screening and prophylaxis (7–9).

As one of the most used RAMs, the Caprini RAM (10) has been applied inside electronic health records in Shanghai Tenth People's Hospital for VTE management of hospitalized patients for several years. In practice, it was performed in a variety of departments at several time points, including admission, before/after surgery, department transfer, and discharge. While it was designed mainly for the surgical population, the validity of Caprini RAM for medical inpatients was also addressed widely and it was suggested to use a single hospital-wide RAM out of practical reasons (11, 12).

With the wide application of artificial intelligence in medical fields, its role in disease prediction, diagnosis, and treatment guidelines has received great attention from clinicians (13). In recent years, a variety of studies have shown that machine learning (ML) algorithms could improve prognostic VTE risk prediction and then help in clinical decision-making. Specifically, ML models were utilized for VTE risk prediction and compared with Khorana RAM among cancer outpatients treated with chemotherapy (14), with Padua RAM among inpatients of the internal medicine (15), with IMPROVE RAM among inpatients (16), and with Caprini RAM among trauma hospitalized population (17). Besides, there have been also many studies focusing on novel or advanced algorithm design based on machine learning and even deep learning techniques. Yang et al. built an end-to-end prediction models from raw electronic medical records based on ontology extraction using natural language process technique (18). Another study designed a hybrid knowledge and ensemble learning method from clinical narratives (19). Some studies developed novel algorithms in a multi-task learning framework to improve the VTE risk prediction for small sample cohorts, such as hierarchical modeling (20) and task-wise split gradient boosting (21). Recently, the theory of neural differential equation was applied to build deep neural competing risk time-to-event VTE models (22).

Although the value of machine learning algorithms in predicting VTE risk has been widely confirmed, most of the studies were based on the clinical indicators of patients during hospitalization or prognosis to build prediction models, which were limited by the feasibility of clinical data. To the best of our knowledge, the problems of ML model development and validation for VTE risk at admission have been seldom studied (8). With data availability for machine learning provided by the filling of Caprini scale at admission, we designed a single-site retrospective study and the research object was to build and evaluate machine learning models based on Caprini variables to assess VTE risk at admission and to explore potential areas of improvement by analyzing a series of model predictive performance.

## Methods

### Study design

We conducted a single-site retrospective study of prognostic prediction modeling at Shanghai Tenth People's Hospital. The clinical data were retrieved from the electronic medical records with complete admission and discharge records between January 2020 and December 2020.

#### Diagnostic criteria of VTE

The two subtypes, DVT and PE, were diagnosed by lower extremity vein ultrasound or venography and chest computed tomography pulmonary angiogram, respectively. Considering that in clinical practice patients might be bedridden or immobilized, we also included bedside ultrasound imaging checks where lower extremity veins were mentioned in the corresponding report texts. Following the same way as our previous study, an imaging check was confirmed as VTE-positive only if positive statements were inspected in the report/conclusion texts, otherwise, it was regarded as negative (20). The VTE check results were additionally confirmed by clinicians for reliability.

#### Study population

Since this study aimed to assess hospital-wide patients' VTE risk status at admission, the cohorts for model development and validation were based on the inpatient departments at Shanghai Tenth People's Hospital where Caprini scale was performed and there were positive VTE patients.

The inclusion criteria were designed as follows: (a) the length of stay at least 3 days, (b) older than 18 years, (c) Caprini scale assessment within 24 h of admission, and (d) at least one VTE imaging check (lower extremity venous ultrasound/venography or chest computed tomography pulmonary angiogram) with clear diagnosis results within 4 days of admission. If there was at least one positive result, the patient was classified into the positive group, otherwise classified into the negative group. A patient was classified into the VTE-positive group if at least one positive imaging check was retrieved.

In consideration of the risk assessment necessity, the following exclusion criteria were introduced: (a) departments where no patients were assigned positive outcomes; (b) patients with positive imaging check results before Caprini scale evaluation within 24 h of admission. In addition, subsequent clinical interventions between Caprini scale and VTE image checking, such as drug prophylaxis and surgery, would induce verification bias and risk status changing on VTE outcomes. We considered two additional exclusion conditions: (c) patients in the positive group who underwent surgery before the positive check record; (d) patients in the negative group who received drug orders (anticoagulant, thrombolytic, and defibrillation drugs) for prophylaxis before the imaging checks.

As a retrospective study, the sample size was determined due to practical rather than statistical considerations. Nevertheless, the values of events per variable were empirically acceptable, and the later experiments showed that model overfitting or underfitting was well controlled.

#### Caprini score and predictors

We extracted the Caprini scale data within 24 h of admission for all patients in the cohort. If there were multiple scale records, then the last one would be used. Briefly, the scale data included a series of risk factors and the final Caprini risk score (CRS). In this study, the risk factors were used as predictors for modeling, while CRS was used for model comparison as the Caprini scale results. According to the study design, the Caprini scales were always performed before VTE image checks to make predictors blind to the outcome and to avoid information leakage.

In clinical practice, the Caprini RAM version 2013 (10, 23) was deployed, which involved 28 risk factors. Among the candidate variables, the variables “*Age*” and “*Bedridden*” were ordinal with four and three levels, respectively, while the rest were binary. The variables could be used for modeling directly without any preprocessing like data imputation since the variables in Caprini scale were completed by clinicians and nurses without any data missing. See Supplementary Table S1 for the full list and abbreviation in this study.

#### Dataset splitting

After cohort establishment, we carried out a stratified random splitting scheme according to the outcome to obtain a training dataset and an independent test dataset, with a size ratio of 7:3. The training dataset was used for the machine learning model development, while the test dataset was used for model validation and performance comparison. Thus, there was no difference between the development and validation datasets in the outcome, predictors, and eligibility criteria.

### Machine learning model development

We conducted machine learning model development based on the training dataset and considered the three most popular binary classification algorithms in clinical predictive modeling tasks: Logistic Regression (LR), Random Forest (RF), and Extreme Gradient Boosting (XGB). For each algorithm, the model training consisted of the following procedures.

#### Hyperparameter tuning

There were some configurations (aka hyperparameters) in each model that should be predefined before training. As a black box optimization problem, hyperparameter search was solved using the Bayesian optimization algorithm. For each model, the optimal hyper-parameters were specified after 200 rounds of searching. See Supplementary Table S2 for hyperparameter range setup.

#### Feature selection

We conducted model-based feature selection after tuning hyperparameters for model reduction. For RF and XGB, we conducted recursive feature elimination (RFE) with 5-fold cross-validation. RFE was a backward feature selection strategy, starting with overall variables, and removing the variable with the least importance (measured by average split gains in any RF or XGB) at every round. The optimal variable subset was acquired by maximizing the cross-validated AUROC values across the whole RFE rounds. For LR, we adopted the LASSO (least absolute shrinkage and selection operator) constraint in the hyperparameter tuning step. After searching the optimal hyperparameters and the variable subset, we retrained plain LR, RF, and XGB on the entire training set again based on the selected features.

#### Calibrating

The calibration of a predictive model for binary outcomes referred to the agreement between outcome probabilities and predictive scores. We performed a univariate Logistic regression (termed Platt scaling) for CRS on the training set to convert the score through a sigmoid function (24). For each machine learning model, an isotonic regression was fitted to adjust the raw model prediction if the hypothesis test for calibration was rejected (24, 25). To avoid overfitting, we took the mean value of the cross-validated isotonic regression outputs as the final probability values.

### Model validation

We assessed the performance of machine learning models as well as CRS in various ways of measures based on the independent test set, including traditional metrics related to discrimination and calibration as well as some novel measures related to reclassification and clinical usefulness (26).

#### Model metrics

As a rule of thumb, we reported the receiver-operating-characteristic (ROC) curves as well as the areas under the ROC curves (AUROC) for all models to measure the primary predictive performance. We also specified the optimal threshold values with respect to maximizing the Youden index and calculated the confusion matrices as well as sensitivity, specificity, and F1 score. Considering the problem of label imbalance, we also presented precision-recall curves (PRC) and the areas under the PRC (AUPRC) as complements for model comparison. For each metric, we conducted statistical inference by means of the nonparametric Bootstrap resampling technique where the number of resampling was set as 5,000. Model calibration was validated through the most popular Hosmer-Lemeshow goodness-of-fit test and the corresponding plots for graphical illustration (27, 28).

The theory of decision curve analysis (DCA) provides a new perspective for model utility evaluation (29–31). To put it simply, assuming that some clinical intervention was performed for individuals greater than a prediction threshold, the benefits of intervention could be evaluated by gains from true positive individuals and losses from false positive ones. Then the decision curves (DCs) were obtained by varying threshold values and computing standardized net benefits. It should be noted that prediction models must be well-calibrated, otherwise DCs would be biased and the comparison between models might be invalid (29).

We used SHapley Additive exPlanations (SHAP) values to measure and visualize the variable contribution and importance in any model (32, 33). As a novel model-agnostic approach for black-box model explanation, SHAP could show how variables participate in model prediction while providing both local personalized prediction interpretability and global variable importance explanation. This method has been widely used in clinical predictive modeling research to improve model transparency (34, 35).

#### Risk stratification application

In real applications, the CRS was categorized into three risk levels for follow-up treatment and nursing. Therefore, the risk stratification of machine learning models was also designed as three levels: low-risk, moderate-risk, and high-risk. The principle of stratification cutoff specification was to keep the false negatives in the low-risk group and the false positives in the high-risk group non-inferior to CRS. For any machine learning model, the low-risk threshold was set up such that the sensitivity corresponded to that of 3 points for CRS, and the high-risk threshold was set up such that the specificity corresponded to that of 5 points for CRS. We then reported some description of stratification results on the test set, including positive rates, level proportion, etc.

We further investigated whether the stratification application of the machine learning models would show any improvement compared to the existing CRS using the net classification improvement (NRI), a measurement defined as the difference between improved and worsened prediction proportions (36). We reported the NRI components of negative and positive groups, respectively. Statistical inference and hypothesis tests were performed using the asymptotic Z statistics.

### Statistical analysis

In the data summary, categorical variables were presented by numbers and percentages, while continuous variables were presented by medians and quantiles. The difference tests between the training and test datasets were implemented by Chi-square, Fisher's exact, and Mann–Whitney *U* test for categorical, binary (with any actual or expected contingency table cell values < 5), and numerical variables, respectively. For any statistic metric we reported the standard derivation (sd) or 95% confidence interval (CI). Statistical significance was defined as *P *< 0.05.

### Code implementation

All the experiments were performed using the Python language (version 3.9). The statistical tests were based on the *scipy* (version 1.9.3) library. The main machine learning development was based on the *scikit-learn* (version 1.1.2) library, including LR, RF, recursive feature elimination, isotonic regression, and main model metrics. XGB, hyperparameter tuning, SHAP explanation were based on the libraries *xgboost* (version 1.6.2), *hyperopt* (version 0.2.7), and *shap* (version 0.41), respectively. The rest computation was implemented manually in the Python environment. The codes for model training and validation were released at https://github.com/WenboSheng/vte-modeling-at-admission.

## Results

### Dataset information

**Figure 1:**
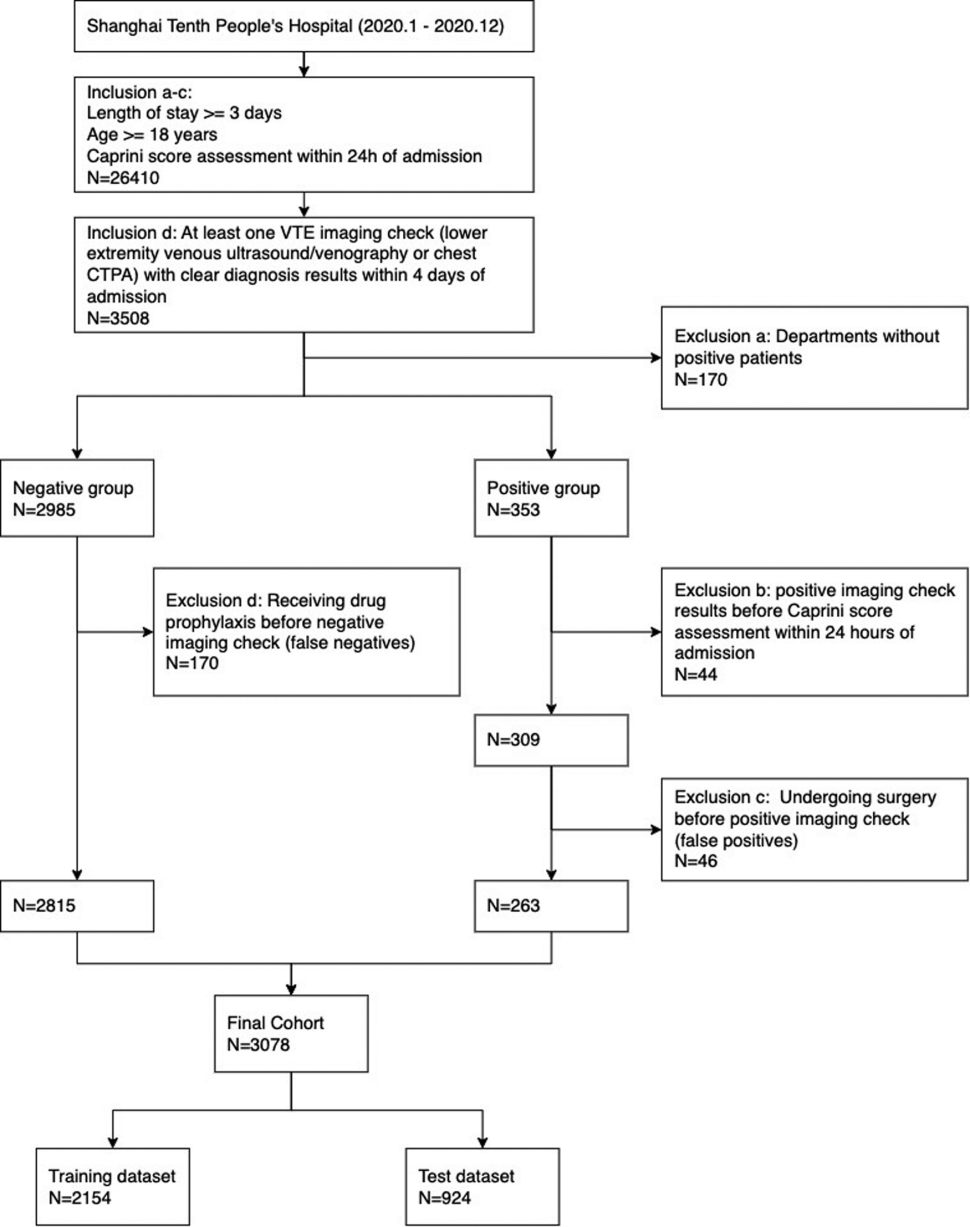
Cohort flowchart.

The cohort in this study included 3,078 individuals from 14 departments, which was divided into a training set and a test set with sample sizes 2,154 and 924. See Figure 1 for the complete participant flow details. The positive rate of the VTE outcome was 8.5%, with 184 and 79 outcome events in the training and test sets, respectively. There was no significant difference between the training set and the test set on the outcome, age, sex, departments, and CRS. See Table 1 for the dataset description.

**Table 1:** Dataset characteristics.

Clinical Features		Overall	Training set	Test set	*P*-Value
*n*		3,078	2,154	924	
Outcome, *n* (%)	Non-VTE	2,815 (91.5)	1,970 (91.5)	845 (91.5)	>0.99
	VTE	263 (8.5)	184 (8.5)	79 (8.5)	
Caprini_risk, *n* (%)	Low (0–2)	1,244 (40.4)	871 (40.4)	373 (40.4)	>0.99
	Moderate (3–4)	859 (27.9)	601 (27.9)	258 (27.9)	
	High (≥5)	975 (31.7)	682 (31.7)	293 (31.7)	
Age, median [Q1, Q3]		64.0 [52.0,73.0]	65.0 [52.0,73.0]	64.0 [52.0,72.0]	0.34
Sex, *n* (%)	Female	1,927 (62.6)	1,366 (63.4)	561 (60.7)	0.17
	Male	1,151 (37.4)	788 (36.6)	363 (39.3)	
Departments, *n* (%)	Cardio-vascular	111 (3.6)	70 (3.2)	41 (4.4)	0.28
	Emergency	19 (0.6)	12 (0.6)	7 (0.8)	
	Endocrinology	431 (14.0)	306 (14.2)	125 (13.5)	
	Geriatrics	17 (0.6)	13 (0.6)	4 (0.4)	
	Gynaecology	734 (23.8)	506 (23.5)	228 (24.7)	
	ICU	95 (3.1)	60 (2.8)	35 (3.8)	
	Neurology	115 (3.7)	79 (3.7)	36 (3.9)	
	Oncology	35 (1.1)	20 (0.9)	15 (1.6)	
	Orthopedics	892 (29.0)	644 (29.9)	248 (26.8)	
	Others^a^	21 (0.7)	15 (0.7)	6 (0.6)	
	Surgery	393 (12.8)	266 (12.3)	127 (13.7)	
	Thyroid&Mammary	114 (3.7)	86 (4.0)	28 (3.0)	
	Traditional Chinese Medicine	29 (0.9)	22 (1.0)	7 (0.8)	
	Urology	72 (2.3)	55 (2.6)	17 (1.8)	

^a^ “Others” included departments of rheumatism immunity, microecology treatment, and blood branch.

### Model prediction performance

The models were fitted on the training set, including hyperparameter tuning, automatic feature selection, and potential calibrating. The final LR selected 15 features (namely, with non-zero coefficients). For RF and XGB, the RFE procedure selected 15 and 14 features, respectively, and additional cross-validated isotonic regressions were necessary since the raw output scores of the two models were found far from well-calibrating. The characteristics of selected variables for RF were presented in Table 2. See Supplementary Table S3 for the details of feature selection as well as model specification information (variable coefficients in LR and feature importance in RF and XGB).

**Table 2:** Variable characteristics included in RF.

Variables^a^		Training set (*n* = 2,154)			Test set (*n* = 924)		
		Negative (*n* = 1,970)	Positive (*n* = 184)	*P*-value	Negative (*n* = 845)	Positive (*n* = 79)	*P*-value
VVV, *n* (%)	0	1,965 (99.7)	168 (91.3)	<0.001	844 (99.9)	73 (92.4)	<0.001
	1	5 (0.3)	16 (8.7)		1 (0.1)	6 (7.6)	
LungDisease, *n* (%)	0	1,945 (98.7)	180 (97.8)	0.305	833 (98.6)	78 (98.7)	1.000
	1	25 (1.3)	4 (2.2)		12 (1.4)	1 (1.3)	
Broken, *n* (%)	0	1,800 (91.4)	159 (86.4)	0.035	776 (91.8)	67 (84.8)	0.057
	1	170 (8.6)	25 (13.6)	69 (8.2)	12 (15.2)		
SwollenLeg, *n* (%)	0	1,966 (99.8)	181 (98.4)	0.017	841 (99.5)	77 (97.5)	0.086
	1	4 (0.2)	3 (1.6)		4 (0.5)	2 (2.5)	
PastMajorSurg, *n* (%)	0	1,914 (97.2)	170 (92.4)	0.001	818 (96.8)	71 (89.9)	0.007
	1	56 (2.8)	14 (7.6)		27 (3.2)	8 (10.1)	
HistoryVTE, *n* (%)	0	1,966 (99.8)	175 (95.1)	<0.001	842 (99.6)	76 (96.2)	0.010
	1	4 (0.2)	9 (4.9)		3 (0.4)	3 (3.8)	
CVC, *n* (%)	0	1,914 (97.2)	161 (87.5)	<0.001	814 (96.3)	67 (84.8)	<0.001
	1	56 (2.8)	23 (12.5)	31 (3.7)	12 (15.2)		
MinSurg, *n* (%)	0	1,868 (94.8)	171 (92.9)	0.359	821 (97.2)	72 (91.1)	0.012
	1	102 (5.2)	13 (7.1)		24 (2.8)	7 (8.9)	
Bedridden, *n* (%)	None	1,736 (88.1)	121 (65.8)	<0.001	730 (86.4)	51 (64.6)	<0.001
	<72 h	86 (4.4)	30 (16.3)			45 (5.3)	14 (17.7)
	≥72 h	148 (7.5)	33 (17.9)			70 (8.3)	14 (17.7)
Stroke, *n* (%)	0	1,805 (91.6)	150 (81.5)	<0.001	773 (91.5)	59 (74.7)	<0.001
	1	165 (8.4)	34 (18.5)	72 (8.5)	20 (25.3)		
IBD, *n* (%)	0	1,963 (99.6)	182 (98.9)	0.176	844 (99.9)	79 (100.0)	1.000
	1	7 (0.4)	2 (1.1)		1 (0.1)		
Obesity, *n* (%)	0	1,748 (88.7)	159 (86.4)	0.411	751 (88.9)	69 (87.3)	0.821
	1	222 (11.3)	25 (13.6)	94 (11.1)	10 (12.7)		
Malignancy, *n* (%)	0	1,706 (86.6)	156 (84.8)	0.565	712 (84.3)	70 (88.6)	0.389
	1	264 (13.4)	28 (15.2)	133 (15.7)	9 (11.4)		
Age, *n* (%)	≤=40 yr	213 (10.8)	5 (2.7)	<0.001	88 (10.4)	2 (2.5)	<0.001
	41–60 yr	595 (30.2)	28 (15.2)			268 (31.7)	13 (16.5)
	61–75 yr	751 (38.1)	86 (46.7)			333 (39.4)	35 (44.3)
	>75 yr	411 (20.9)	65 (35.3)	156 (18.5)	29 (36.7)		
MajorSurg, *n* (%)	0	1,913 (97.1)	172 (93.5)	0.014	814 (96.3)	68 (86.1)	<0.001
	1	57 (2.9)	12 (6.5)		31 (3.7)	11 (13.9)	

^a^VVV, visible varicose veins; PastMajorSurg, past major surgery (>45 min) within last month; HistoryVTE, history of blood clots, either DVT or PE; CVC, tube in blood vessel in neck or chest that delivers blood or medicine directly to heart within the last month; MinSurg, minor surgery (<45 min) is planned; Bedridden, on bed rest or restricted mobility, including a removable leg brace for less than 72 h; IBD, a history of inflammatory bowel disease; Malignancy, current or past malignancies (excluding skin cancer, but not melanoma); MajorSurg, length of a surgery over 2 h. See Supplementary Table S1 for the full description.

**Figure 2:**
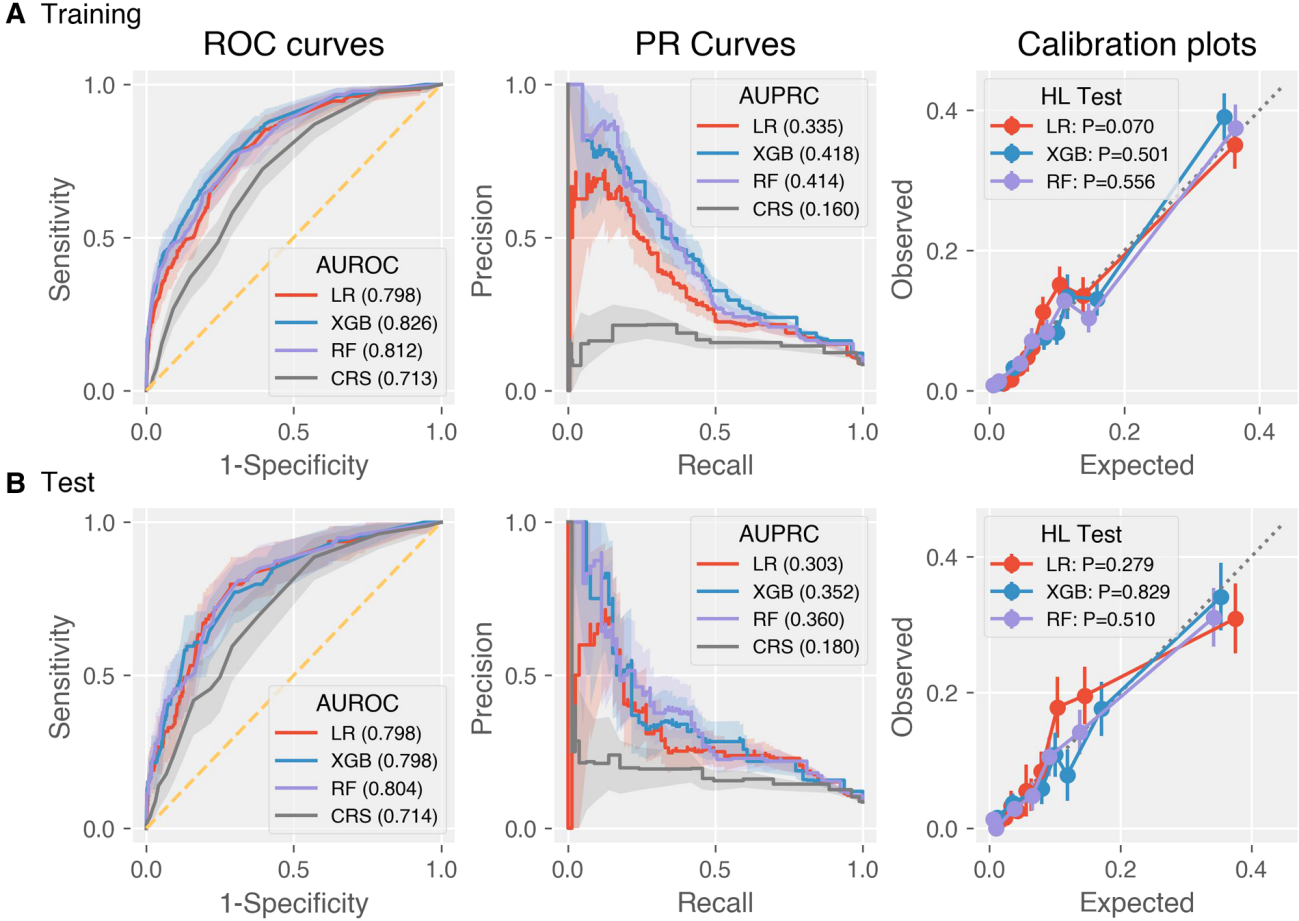
Discrimination and calibration performance of CRS and the three machine learning models LR, XGB, RF on the (**A**) training and (**B**) test datasets. Left panel: Receiver operating characteristics curves (legends: Area under ROC of each model); middle panel: precision-recall curves (legends: Area under PRC of each model); right panel: calibration plots (legends: *P*-values of Hosmer-Lemeshow tests).

The discrimination metrics were illustrated in Table 3, including AUROC, AUPRC, sensitivity, specificity, precision, and F1 score. Note that sensitivity, specificity, precision, and F1 score were based on the optimal Youden index. The ROC curves of the predictive models were shown in Figure 2, left panel. It can be concluded that the AUROC values of all the machine learning models are significantly superior to CRS (*P *< 0.001, pairwise comparison with Bonferroni correction). Besides, RF achieved the best score (AUROC = 0.804, 95% CI 0.750–0.852), whereas the difference among the three machine learning models was not significant (*P *= 0.60). Due to the label imbalance, we used PRC and AUPRC as the secondary assessment index, which paid more attention to the prediction of minor labels (Figure 2, middle panel). Also, all the LR, RF, and XGB outperformed CRS significantly (*P *= 0.003). Besides, RF achieved the best score (AUPRC = 0.630, 95% CI 0.268–0.464), and was higher than LR significantly (*P *= 0.015). The calibration curves were displayed in Figure 2, right panel. All the Hosmer-Lemeshow tests were not significant (*P* values larger than 0.05), indicating the consistency between prediction and observation on both training and test sets for all the models (RF and XGB were adjusted by isotonic regression).

**Table 3:** Prediction performance with 95% CI of the investigated models on the test set.

	CRS	LR	XGB	RF
AUROC	0.714 (0.656, 0.767)	0.798 (0.745, 0.845)	0.798 (0.745, 0.846)	0.804 (0.750, 0.852)
AUPRC	0.180 (0.133, 0.247)	0.303 (0.227, 0.415)	0.352 (0.261, 0.452)	0.360 (0.268, 0.464)
Sensitivity	0.886 (0.595, 0.797)	0.797 (0.709, 0.886)	0.772 (0.671, 0.861)	0.810 (0.722, 0.886)
Specificity	0.431 (0.585, 0.650)	0.715 (0.646, 0.708)	0.701 (0.669, 0.730)	0.688 (0.656, 0.718)
Precision	0.127 (0.124, 0.167)	0.207 (0.166, 0.210)	0.194 (0.170, 0.219)	0.195 (0.173, 0.218)
F1	0.222 (0.205, 0.275)	0.329 (0.270, 0.338)	0.310 (0.272, 0.347)	0.314 (0.280, 0.349)

The cutoff values for sensitivity, specificity, precision, and F1 were derived from the maximal Youden's indexes.

**Figure 3:**
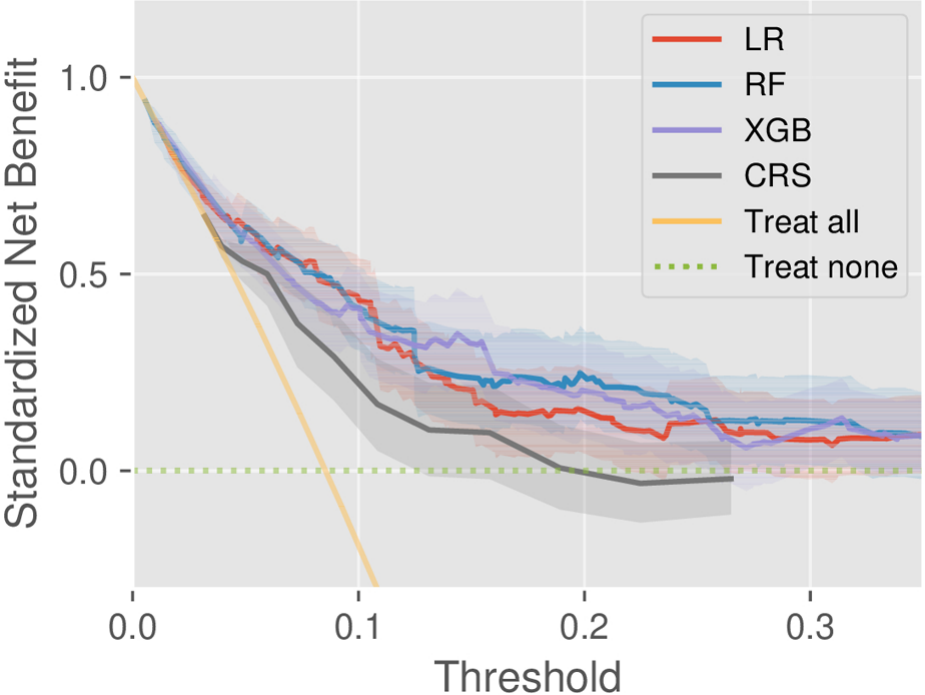
Decision curve analysis of the models CRS, LR, RF, and XGB, as well as two references (treat-all and treat-none).

We investigated the clinical utility through DCA on the test set. According to the DCA theory, there are two reference curves by default, that is, the no intervention (treat none) and all intervention (treat all) scenarios. As shown in Figure 3, the machine learning models provided greater net benefit values than CRS at almost all risk thresholds. In addition, the decision curve of RF is superior to that of LR, but the comparison results between RF and XGB depend on threshold specification. Besides, the model validity was confirmed by the fact that all the DCs were completely located upon the two reference curves.

**Figure 4:**
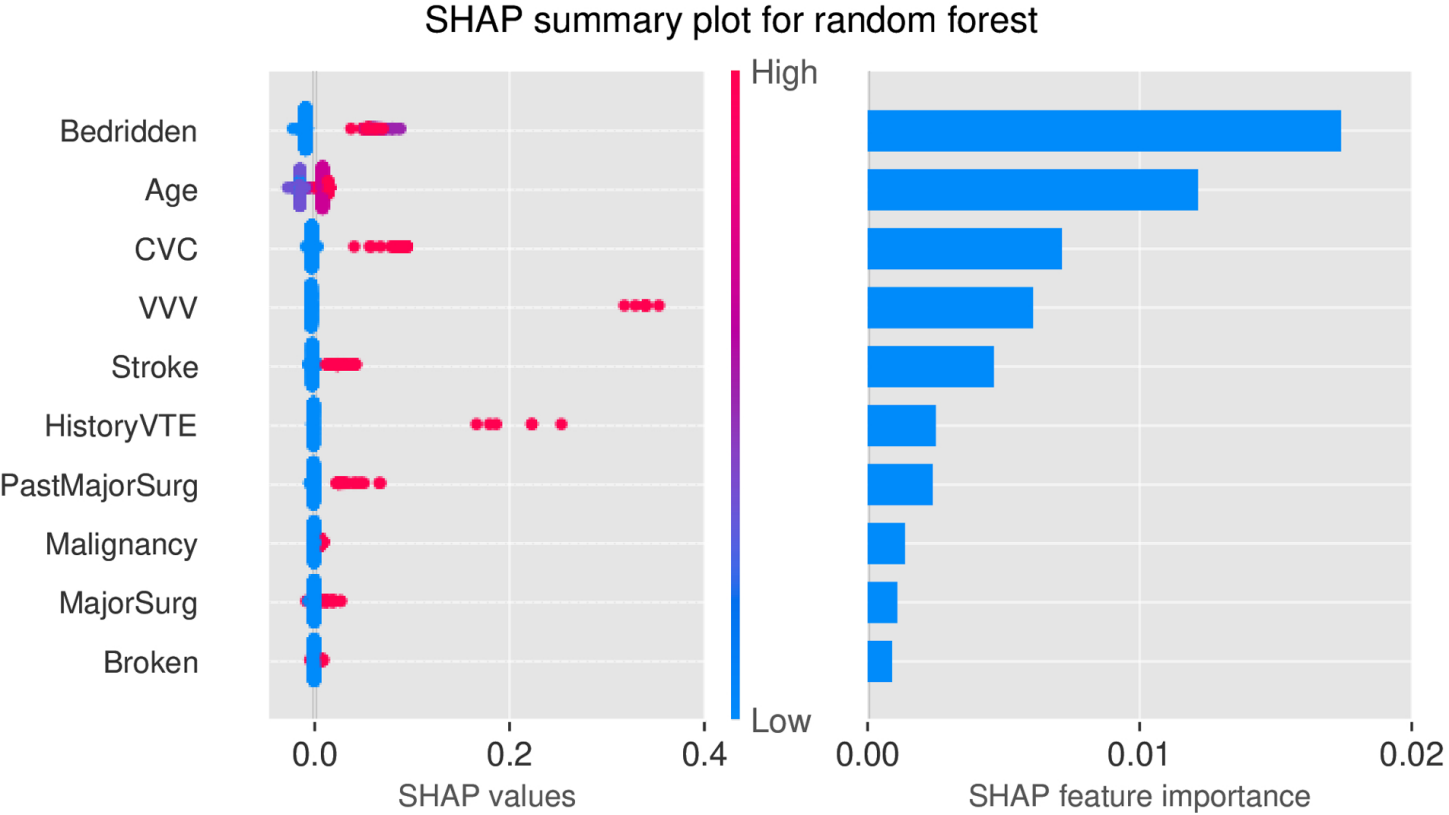
SHAP plots for RF. Left: SHAP values of top 10 variables; Right: Feature importance of top 10 variables. Bedridden—on bed rest or restricted mobility, including a removable leg brace for less than 72 h; CVC—tube in the blood vessel in neck or chest that delivers blood or medicine directly to the heart within the last month; VVV—visible varicose veins; HistoryVTE - history of blood clots, either DVT or PE; PastMajorSurg - past major surgery (>45 min) within last month; Malignancy—current or past malignancies; MajorSurg—length of a surgery over 2 h; Broken—broken hip, pelvis, or leg.

Figure 4 illustrated the SHAP values of RF. As shown in the right panel of Figure 4, the top five important factors considered by RF for prediction were bedridden (on bed rest or restricted mobility, including a removable leg brace for less than 72 h), age, CVC (tube in the blood vessel in neck or chest that delivers blood or medicine directly to the heart within the last month), VVV (visible varicose veins), and stroke (experienced a stroke). As included in Caprini scale, these risk factors have been confirmed with VTE by many previous studies. In the SHAP value plot (Figure 4, left panel), all variables had a positive relationship with risk, which also verified a valid clinical implication of RF.

### Risk stratification

**Figure 5:**
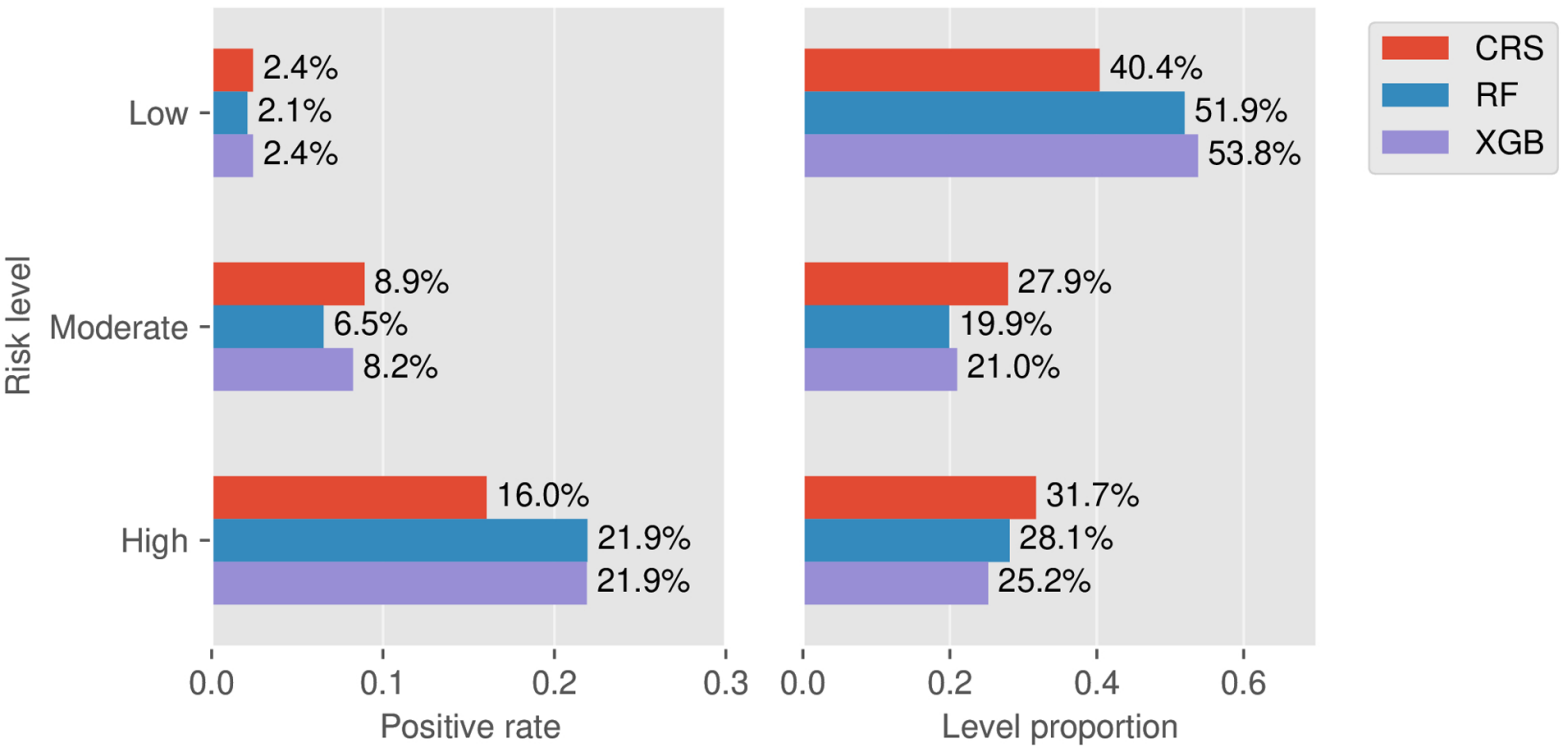
Stratification results. Left: Rates of positive individuals in the three stratified levels for the models CRS, RF, and XGB. Right: Proportion of the three stratified levels for the models CRS, RF, and XGB.

Taking 3 points and 5 points as the two thresholds in CRS, the false negative rate (1-sensitivity) in the low-risk group and the false-positive rate (1-specificity) in the high-risk group on the training set were 13.0% and 29.2%, respectively. Then the two values were used as a reference for risk stratification of RF and XGB as described in Figure 5. Here we did not include LR since its prediction performance was weaker than RF from the aforementioned AUPRC and DCA. Taking RF vs. CRS as an example, the positive rate of the low-risk group decreased from 2.41% to 2.08%, and the positive rate of the high-risk group increased from 16.0% to 21.9%. The proportion of the low-risk group increased from 40.4% to 51.9%, and the proportions of the moderate- and high-risk groups decreased from 27.9% to 19.9% and from 31.7% to 28.1%, respectively. This indicated a higher risk enrichment ability for the machine learning model than CRS.

Further, we carried out NRI analysis to investigate the improvement of RF and XGB against CRS. The NRI components for the positive and negative groups of RF were 0.101 (sd = 0.046, *P *= 0.03) and −0.135 (sd = 0.016, *P *< 0.001), respectively. The statistical inference showed that RF could correct those misclassified individuals effectively. In contrast, for XGB we found a significant negative NRI value of −0.17 (sd = 0.017, *P *< 0.001) but a non-significant positive NRI value (NRI_positive = −0.01, *P *= 0.81). See Supplementary Table S4 for the detailed reclassification results across the risk levels. The NRI analysis showed that RF might have a better ability for prediction improvement than XGB.

### Sensitivity analysis

In the cohort design of this study, VTE risk at admission was investigated by means of occurrence within 4 days of staying since the length of stay itself was considered a risk factor of VTE. To indicate the rationality of the setting, we modified this parameter to 7 and 15 days to explore the predictive effect on medium- and long-term VTE risk during hospitalization. The steps of cohort construction, variable and outcome setup, and machine learning modeling followed the same design protocol. It could be concluded that the predictive performance weakened slightly with the increase of the time span parameter. Nevertheless, the conclusion that machine learning models outperformed CRS always remained. See Supplementary Figure S1 for the results of AUROC trends on the test sets.

## Discussion

Early screening within 24 h at admission was an important issue for VTE management during hospitalization. In this single-site study, we established machine learning models for risk assessment at admission and evaluated the corresponding prediction performance and potential improvement for the Caprini score.

We considered LR, a traditional linear model, as well as RF and XGB, the two most popular tree-based models in biomedical classification tasks. As displayed in Figure 2, the AUROC and AUPRC values of each model on the training and test datasets were similar, which implied that the pitfalls of overfitting or underfitting were well controlled during model training. In summary, machine learning models could elevate the accuracy of risk assessment effectively compared to CRS. By means of a comprehensive analysis of evaluation metrics including AUROC, AUPRC, DCA, and NRI, RF was found to achieve the best prediction performance. The ML models could be potentially used together or in place with Caprini scale at admission to provide more information on VTE risk for inpatients from surgery, orthopedics, and many other departments. Of course, more external model-validation studies were necessary before actual clinical practice for such newly developed models.

There are several problems with RAMs and machine learning models in previous studies. First, clinical variables collection was cost-consuming, including variable design, data extraction, quality control, feature engineering, etc. The cost was proportional to the clinical variable number, which limited the applicability of machine learning models, especially for those complex models that involved a lot of variables. Second, the timing of availability was a critical issue in the admission scenario. Many indicative clinical variables in the previous study were infeasible before Caprini scale at admission, especially the time-aware variables like in-hospital duration and the blood coagulation-related indicators from specialized blood biochemical tests (37). Inhospital duration values were always within 24 h and hence made no sense. We found that the missing rates of the coagulation variables such as D-dimer, C-reactive protein, fibrinogen, and prothrombin at admission before Caprini scale were large and generally regarded unacceptable in modeling. Such hospital-wide systematic data missing might result from the diversity of diagnosis and treatment pathways among different departments. In addition, it was unfair and of limited validity to make comparison between machine learning models and CRS if they stemmed from different regimens of variable inclusion. In contrast, relying on the Caprini variables in this study could get rid of these problems. No additional variables were required to be designed and extracted. The same variable scheme used by both CRS and machine learning models ensured the data completeness, clinical interpretability, and plausibility of model comparison.

During model training, we introduced the mechanisms of hyperparameter tuning and feature selection for optimization and avoiding overfitting. Feature selection could reduce model complexity and improve the robustness and generalization ability. Involving too many variables may both increase the risk of overfitting and lessen model interpretability, and hence hinder the clinical application. In this study, we used a model-based stepwise selection approach, which tried to search the most suitable feature subset for each model in a greedy search manner, and was popular in clinical prediction modeling (38). The results show that using fewer variables could achieve better or non-inferior results in machine learning models.

In this study, the positive rate was 8.5%, a typical case of mild class imbalance. We introduced PRC to better focus on the classification of the positive samples. In general, while the ROC curve and AUROC described the overall discriminative ability, PRC and AUPRC were known to be more informative for class-imbalanced predictive tasks (39). We found that despite no significant difference for AUROC among machine learning models, RF achieved the best score of AUPRC and was significantly higher than LR. This revealed that RF could learn the feature information of the VTE-positive class more effectively, and hence harbored better predictability. This also demonstrated that PRC was an effective supplementary indicator for model comparison and selection in imbalanced problems. This conclusion was also supported by the DCA results.

SHAP interpretation was a novel and powerful tool to analyze feature contribution in any machine learning model. Compared with traditional tree importance or permutation importance, SHAP could not only provide overall feature ranking but also reveal the positive/negative relationships between variables and the outcome. The top five variables identified from SHAP explanation were bedridden, age, central venous catheter, visible variable veins, and stroke. All these variables were confirmed to affect VTE risk and have positive clinical significance for screening and prevention by several studies. A clinical study confirmed that acute stroke, age ≥70 years old, and bedridden were independent risk factors for the occurrence of DVT (40). Central venous catheter insertion could cause local venous damage at the entry site, leading to decreasing in peripheral blood flow and thereby increasing the risk of thrombosis (41). It was also found that VTE risk increased among population with variant veins (42).

Many predictive models were evaluated only from the algorithmic perspective, such as the above-mentioned ROC, PRC, and Youden index. We additionally considered the clinical application validity. Following the usage of Caprini scale, we performed risk stratification (low-, moderate-, and high-risk levels). The setup of cutoff values was a subtle issue and entailed careful research since too high or too small cutoff values would lead to low sensitivity or specificity. The analysis results showed that the machine learning model has a higher risk enrichment ability than CRS. At the low-risk level, the increased sample size and decreased positive rates could relieve the missed diagnosis and medical care costs. At the high-risk level, the reduced false alarm rate could improve the acceptance of medical staff to use this tool. Besides, at the moderate-risk level, the decreased sample size could reduce the model prediction ambiguity.

We further analyzed the improvement of the machine learning model using NRI. At the output setup of three levels, the prediction improvement was defined as upward levels for positive or downward levels for negative. Previous studies have shown that the overall additive NRI index has serious criticism and controversy such as misleading, false positives, and lack of reasonable explanation (43–45). Hence, we reported the NRI components rather than the overall NRI (46). The results showed that RF was effective in correcting the misclassification by CRS in both the positive and negative groups.

This study has some limitations. First, this study is a single-site retrospective study with inevitable underlying confounding factors and bias, and the results have not been prospectively or externally verified. External validation was necessary to investigate whether the ML models outperformed the Caprini score in clinical practice. It is particularly noteworthy that most of hospitalized patients were in a low VTE risk state with absence of imaging checks, and the risk stratification and prediction performance of ML models in this group urgently need to be explored. Second, this study is based on Caprini variables. But the clinical pathway for a patient to receive Caprini scale or not was not in consideration. Such inclusion bias would interfere with cohorts and data distribution, and thus would limit the generalization of the follow-up data-driven models. Lastly, we simply excluded patients if an intervention was found before image checking (golden criteria) since such intervention would change VTE risk and the outcome of image checking could not reflect the risk status at admission. Such verification bias might be further improved, for instance, in the framework of semi-supervised learning. Somehow, this study implied that machine learning techniques could be used for VTE risk assessment at admission and boost prediction performance. More model development and validation studies should be further conducted to promote clinical applications.

## Conclusion

In conclusion, machine learning algorithms could be utilized to build models to assess VTE risk at admission effectively. Based on the variables of Caprini scale, the prediction performance of the random forest model for VTE was demonstrated superior to both other machine learning models and Caprini scores.

## Data Availability

The original contributions presented in the study are included in the article/**Supplementary Material**, further inquiries can be directed to the corresponding author.
