# Supplementary material for: Development and validation of machine learning models for venous thromboembolism risk assessment at admission: a retrospective study
Source: Front Cardiovasc Med. 2023 Aug 29;10:1198526. doi: 10.3389/fcvm.2023.1198526 (PMC10497101; doi:10.3389/fcvm.2023.1198526)
Supplement: Supplementary file 1 [file Datasheet1.docx]

Supplementary Appendix for “Development and validation of machine learning models for venous thromboembolism risk assessment at admission: a retrospective study”

Contents

[Supplementary Tables 2](#_Toc124263916)

[Supplementary Figures 6](#_Toc124263917)

# Supplementary Tables

Table S1: List of Caprini variables and scores

| Abbreviation | Caprini variables | Score |
| --- | --- | --- |
| AdvPregnancy | history of unexplained stillborn infant, recurrent spotaneous abortion (more than 3), premature birth with toxemia or growth restricted infant | 1 |
| BC&HRT | current use of birth control or hormone replacement therapy | 1 |
| CHF | congestive heart failure | 1 |
| HeartAttack | heart attack | 1 |
| IBD | a history of inflammatory bowel disease | 1 |
| LungDisease | lung disease | 1 |
| MajorSurg | length of a surgery over 2 hours | 1 |
| MinSurg | minor surgery (<45min) is planned | 1 |
| Obesity | overweight or obese | 1 |
| PastMajorSurg | past major surgery (>45 min) within last month | 1 |
| Pregnancy&Postpartum | pregnant or had a baby within the last month | 1 |
| SeriousInfection | serious infection | 1 |
| SwollenLeg | swollen legs (current) | 1 |
| VVV | visible varicose veins | 1 |
| CVC | tube in blood vessel in neck or chest that delivers blood or medicine directly to heart within the last month | 2 |
| Immobilization | non-removable plaster cast or mold than has kept you from moving your legs within the last month | 2 |
| Malignancy | current or past malignancies (excluding skin cancer, but not melanoma) | 2 |
| PlanMajorSurg | planned major surgery lasting longer than 45min (including laparoscopic and arthoscopic) | 2 |
| FamilyVTE | Family history of blood clots | 3 |
| HistoryVTE | history of blood clots, either DVT or PE | 3 |
| PositiveBlood | Personal or family history of positive blood test indicating an increased risk of blood clotting | 3 |
| Broken | broken hip, pelvis, or leg | 5 |
| SeriousTrauma | serious trauma | 5 |
| SpinalCordInjury | spinal cord injury resulting in paralysis | 5 |
| Stroke | experienced a stroke | 5 |
| THA&TKA | elective hip or knee joint replacement surgery | 5 |
| Age | age | † |
| Bedridden | on bed rest or restricted mobility, including a removable leg brace for less than 72 hours | ‡ |

† 1 for 41-60 years; 2 for 61-74 years; 3 for 75 years or over.

‡ 1 for bed rest <= 72 hours; 2 for bed rest > 72 hours.

Table S2: Hyperparameter setup

| Model | Hyperparameters | Range |
| --- | --- | --- |
| LR with LASSO | C | 0.01--10 |
| RF | max_depth | 2--8 |
|  | n_estimators | 20--100 |
| XGB | n_estimators | 20--100 |
|  | learning_rate | 0.001--0.1 |
|  | max_depth | 2--8 |
|  | reg_lambda | 0.01--10 |
|  | reg_alpha | 0.01--10 |
|  | subsample | 0.6--1 |
|  | gamma | 0--20 |

Table S3: Variable selection and model details

| Variables | LR_Coef † | RF_Feat_Imp | XGB_Feat_Imp |
| --- | --- | --- | --- |
| Bedridden | 0.4741 | 0.18 | 0.1137 |
| Age | 0.653 | 0.0883 | 0.0462 |
| THA&TKA | -0.2944 | / | 0.0415 |
| Stroke | 0.4031 | 0.043 | 0.0269 |
| SpinalCordInjury | / | / | / |
| SeriousTrauma | / | / | 0.0321 |
| Broken | 0.3058 | 0.0103 | 0.0286 |
| PositiveBlood | / | / | / |
| HistoryVTE | 3.3327 | 0.0953 | 0.1609 |
| FamilyVTE | / | / | / |
| PlanMajorSurg | 0.5858 | / | 0.0272 |
| Malignancy | 0.3726 | 0.0182 | 0.0273 |
| Immobilization | / | / | / |
| CVC | 1.2028 | 0.1102 | 0.046 |
| VVV | 4.3544 | 0.2457 | 0.3382 |
| SwollenLeg | 1.6681 | 0.0235 | / |
| SeriousInfection | / | / | / |
| Pregnancy&Postpartum | / | / | / |
| PastMajorSurg | 0.8942 | 0.0344 | 0.0309 |
| Obesity | 0.4337 | 0.0114 | 0.0488 |
| MinSurg | 0.2884 | 0.0168 | / |
| MajorSurg | 0.2399 | 0.0187 | 0.0316 |
| LungDisease | / | 0.0057 | / |
| IBD | / | 0.0065 | / |
| HeartAttack | / | / | / |
| CHF | / | / | / |
| BC&HRT | / | / | / |
| AdvPregnancy | / | / | / |

Abbreviation. LR_Coef: linear coefficients in the LR model; RF_Feat_Imp: feature importance values in the RF model; XGB_Feat_Imp: feature importance values in the XGB model.

† The intercept of LR was -4.3873.

Table S4: Reclassification details

|  | Group | Positive | | | Negative | | |
| --- | --- | --- | --- | --- | --- | --- | --- |
|  | Level | **Low** | **Moderate** | **High** | **Low** | **Moderate** | **High** |
|  | RF vs CRS | | | | | | |
| CRS | Low | 7 | 1 | 1 | 357 | 0 | 7 |
|  | Moderate | 3 | 11 | 9 | 71 | 132 | 32 |
|  | High | 0 | 0 | 47 | 42 | 40 | 164 |
|  | XGB vs CRS | | | | | | |
| CRS | Low | 7 | 1 | 1 | 352 | 11 | 1 |
|  | Moderate | 6 | 11 | 6 | 94 | 114 | 27 |
|  | High | 2 | 1 | 44 | 56 | 36 | 154 |

# Supplementary Figures

Figure S1: AUROC trending for different VTE risk periods
